# Patterns of self-medication with antibiotics in Maputo City: a qualitative study

**DOI:** 10.1186/s13756-019-0618-z

**Published:** 2019-10-21

**Authors:** Neusa F. Torres, Vernon P. Solomon, Lyn E. Middleton

**Affiliations:** 1 Instituto Superior de Ciências de Saúde (ISCISA), High Institute for Health Sciences, Maputo, Mozambique; 2 Discipline of Public Health Medicine, School of Nursing and Public Health, College of Health Science, University of Kwazulu-Natal, Durban, South Africa; 3 Discipline of Pharmaceutical Sciences, College of Health Sciences, University of KwaZulu-Natal, Training for Health Equity Network (THEnet), Durban, South Africa

**Keywords:** Self-medication, Patterns, Non-prescribed utilization, Antibiotics, Maputo City

## Abstract

**Background:**

Mozambique classifies but does not yet enforce antibiotics as prescription-only-medicine (POM) allowing the public access to a variety of antibiotics that otherwise are provided on prescription. This contributes to the growing practice of self-medication with antibiotics (SMA) which systematically exposes individuals to the risk of developing antibiotic resistance, antibiotic side effects and increases the health service costs and morbidity. This study aimed at describing the patterns of SMA among Maputo city pharmacy customers.

**Methods:**

A qualitative study conducted between October 2018 and March 2019 was developed with thirty-two pharmacy customers and seventeen pharmacists. Using convenience sampling, customers were recruited after buying antibiotics without prescription from nine private pharmacies. Of the thirty-two participants, twenty participated in in-depth interviews and twelve in two focus groups discussions (FGD) with six participants each. Purposive sampling and a snowball technique were used to recruit pharmacists. The transcripts were coded and analyzed using latent content analysis. Nvivo 11 was used to store and retrieve the data. The COREQ (Tong, 2007) checklist for interviews and FGD was performed.

**Results:**

Customers admitted practices of SMA, pharmacists admitted dispensing a variety of antibiotics without prescription. Non-prescribed antibiotics (NPA) were obtained through five different patterns including; using the generic name, describing the physical appearance and using empty package, describing symptoms or health problem to pharmacists, using old prescriptions and sharing antibiotics with family, friends, and neighbors.

**Conclusion:**

Different patterns of SMA are contributing to the indiscriminate use of antibiotics among customers. The NPA utilization is perceived as an expression of self-care where participants experience self-perceived symptoms and indulge in self-treatment as a method of caring for themselves. Moreover, antibiotics are mostly used to treat diseases that do not necessarily need antibiotics. Strong and effective public health education and promotion initiatives should be implemented to discourage inappropriate utilization of antibiotics and SMA practices.

## Background

Self-medication with antibiotics (SMA) is considered one chief factor driving inappropriate utilization of antibiotics which is closely related to the emergence of antimicrobial-resistant strains [1–4]. Globally, SMA represents an important public health issue as it exposes the individual to health risks such as the development antimicrobial resistance, misdiagnosing illnesses, wrong therapeutic indication, dangerous adverse drug reaction, and drug interactions. It also causes delays seeking medical attention when needed what consequently increases morbidity and/or mortality as well as the costs of health-care services acquiring potent antibiotics to fight resistant infections [5]. Antibiotic resistance is the major concern of the post-antibiotic era, bearing in mind the downturn in the development of new antimicrobials in the pharmaceutical industry that creates unexpected challenges in the effective management of infections [6].

The practice of SMA is common worldwide, with more occurrence in Low-and-Middle-Income Countries (LMICs) where governments are paying high prices to purchase medicines [7]. Despite being prescription-only medicines (POM), antibiotics can be purchased without a valid prescription in many LMICs [8, 9]. According to the WHO, over 50% of global antibiotic prescriptions are inappropriate with 2/3 of antibiotics available in the pharmaceutical market being used for self-medication [10]. After analgesics, antibiotics are the commonly used drugs for self-medication globally, with over 50% purchased and used without prescription [11]. The practices of SMA are reported to be considerably high with European countries recording highest rates within the southern Europe countries such as Greece (20%), Romania (16%), and (14%) for Cyprus. In contrast, other European countries such as Sweden (2%) and Slovakia (3%) have the lowest rates [4]. Yet, a recent systematic scoping review of 31 studies conducted in the USA reported frequent practices of SMA were the prevalence of non-prescription antibiotic use varied from 1 to 66% [12]. In LMICs such as Kuwait, prevalence rates registered were as high as 92% [13]. Moreover, the statistics for SMA in Africa ranges from 24 to 76% with Northern Nigeria at 50.3%, Sierra Leone 68.9%, Ghana 70%, Uganda 65.1%, and Sudan at 76% [14].

The literature reporting the trends of non-prescribed antibiotic (NPA) use by the public in Mozambique is scarce. Nevertheless, some studies reported the irrational use of antibiotics by the community and health care providers [15, 16]. The country report on the Situation Analysis conducted by the Global Partnership for Antibiotic Resistance (GARP), in 2015, noticed high consumption of antibiotics at the community level, with people buying and consuming antibiotics without seeking advice from a qualified health care professional (HCP) and or without a medical prescription [17]. The report adds that despite the existent law and regulation on medicine use, antibiotics are commercialized with insufficient control, added to the explosion of private pharmacies within the capital cities being the main stage of such commercial trends. This adversely paves the way to the easy access to antibiotics and their inappropriate use, which consequently increases resistant infections, morbidity and mortality rates [17].

Whereas patterns of SMA are defined in this study as the way people request, purchase or acquire antibiotics for self-diagnosed diseases, NPA are defined as the use of antibiotics to treat self-diagnosed diseases without a valid prescription. According to Foster and Bandawe (2014), seeking NPA in the pharmacy, sharing with family members, relatives or friends, keeping the empty package for future events of sickness are examples of patterns of SMA [18]. Yousef et al., (2008), Kamat and Nichter (1994) and Saengcharoen et al., (2008), summarized the common patterns of SMA according to the research conducted on the uses of medicines in a range of settings and contexts. Those authors mentioned the following common patterns:

- Direct self-medication, where patients may order non-prescription drugs either by their scientific/generic names, brand names or physical appearance.
- Direct self-medication, where patients use leftover antibiotics from previous disease events and don’t seek professional advice for health problems;
- Direct self-medication where patients order the drug by presenting the empty previous drug package.
- Indirect self-medication, where patients seek the advice of a pharmacy staff regarding their ailments before purchasing the needed medicine.
- Indirect self-medication, where patients seek advice regarding their ailments or share antibiotic with family members, friend or relative before receiving the antibiotic.

The patterns of SMA were found to vary across socioeconomic groups [19], according to the socio-economic, cultural contexts and also according to the efficiency of the health care system. As mentioned above, studies have documented high prevalence’s rates of SMA in some LMICs including in many African countries. Therefore, the patterns in which customers acquire, request or purchase NPA in Maputo city are worth investigating and understanding to provide evidence-based information and identify the diverse factors driving the practices of SMA. This is thus crucial to better address the strategies to promote the rational utilization of antibiotics and improve the antibiotic stewardship and conservancy.

Investigating and documenting the patterns of SMA, the “how” and “why” people practice SMA represents a key way to generate evidence to better inform decision and strategies to improve the adequate utilization of antibiotics at community and individual level. More than quantitative information, qualitative and comprehensive evidence-based information are vital to understanding the reasons behind the practices of SMA. This has the potential to ascertain the appropriate kind of intervention that would focus on the barriers, challenges, and gaps in health promotion and education.

### Aim and research question

- This study aims at describing the patterns of SMA in Maputo city.

The interest was on the forms, models or patterns that customers of pharmacies use to access, request or purchase NPA in Maputo city. The research question that guided this study was:

- What are the patterns of SMA amongst Maputo city pharmacies customers?

## Methods

### Study design and setting

This study uses a qualitative descriptive approach to develop a deep understanding of the complexity of the phenomenon of SMA. Data collection occurred between October 2018 to March 2019 in nine private pharmacies from three socio-economic areas (high, middle and low) of Maputo, the capital city of Mozambique.

### Study participants

Two categories of participants were included: the pharmacy customers and the pharmacy workers (pharmacists). All participants were residents of Maputo city and spoke Portuguese, the official language of Mozambique.

While the inclusion of customers was based on the need to describe the practice of SMA to understand the models in which customers request NPA, the inclusion of pharmacists was based on the need to enrich the data and capture the patterns of SMA based on the perspectives of the dispensers of antibiotics.

### Sampling and recruitment strategy -customers

There are more than sixty pharmacies in the city scattered in three different socio-economic areas. To ensure that pharmacies from the three areas were included, three pharmacies were randomly selected from each socio-economic area and a list of nine was compiled and approached. Using purposeful sampling strategy; pharmacy customers were face-to-face approached when exiting the pharmacy. All customers who purchased any medicine between 8:00 am to 6:00 pm during the above-mentioned period and could not provide a valid prescription^1^ were cordially invited to show up their medication purchases. If medicine purchased was an antibiotic in a form of tablets, capsules or pills, drops, cream/ointment or syrup, customers were invited to participate. Customers less than 18 years old and those purchasing antibiotics on behalf of someone else were excluded.

Overall eighty-four customers were approached in total with forty-four having valid prescriptions and forty without a valid prescription of which eight refused to participate, while twenty gave consent to participate in individual interviews, twelve consented to participate in the FGD. Followed by in-depth interviews, two FGD was held with six participants each. Participants from FGD were different from the interviewed individually. The duration of the interviews varied from twenty to forty-eight minutes, and the two FGD lasted forty-five and fifty minutes.

### Sampling and recruitment strategy for pharmacists

There are two levels of training for the pharmacy profession in Mozambique; a) in a health sciences training institution namely the pharmacy technician^2^ (which comprehends a medium level) and in a university, namely the pharmacist^3^ (which has a university degree in pharmacy). Both categories of professionals are called pharmacy workers, their activities at the pharmacy vary from managing the pharmacy and the drug stocks to the dispensing of medicines. In this study, we adopted the term pharmacist to refer to pharmacy professionals regardless of the level of pharmacy training.

Nineteen pharmacy professionals from the selected pharmacies were purposively contacted with seventeen agreeing to participate. Two pharmacists dropped due to time constraints. First, three of the seventeen were recruited by the phone, their phone details were solicited from a key person from the National Department of Pharmacy (NDP) at the Ministry of Health (MoH). Fourteen were recruited using a snowball method. The duration of interviews varied from fifteen to thirty-eight minutes.

### Ethical approval

All participants were given covering letters explaining the purpose of the study and assuring the confidentiality of information. Participants signed the informed consent to participate and to audio-record the interviews. No name was collected and registered for customers. Pharmacy professional’s names were inevitable to collect since they were first approached by the phone. All names were concealed to guarantee the confidentiality of participants. Ethical clearance was sought both at the University of KwaZulu Natal and at the Health National Bioethics Committee of Mozambique. **Ethical Approval Number:** HSS/0142/018D-UKZN South Africa, 376/CNBS/18 - MoH Mozambique.

### Data collection and study tools

**Fig. 1:**
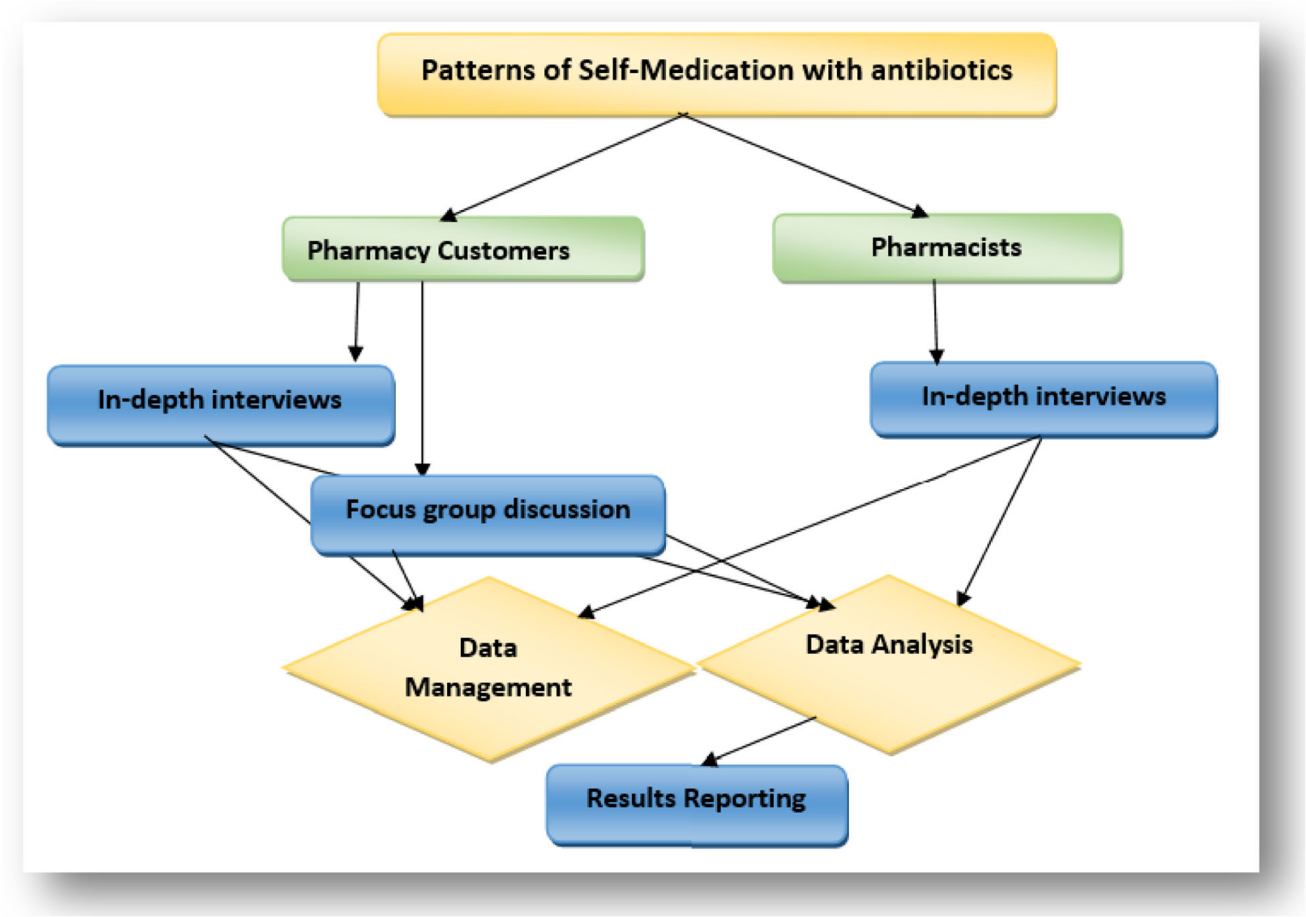
Data collection and analysis flow chart

Figure 1. shows the data collection and analysis flow chart. Primary data were collected by the main author and two Pharmacy graduated research assistants using face-to-face in-depth interviews and focus group discussions with open-ended questions. Interviews and FGD were audio-recorded. Semi-structured interviews allow for structure, flexibility, and flow, ensuring that the interviewer addresses the research questions in full, prompting and probing respondents for further information where necessary. They also allow the respondent to feel engaged in a conversation rather than answering a structured survey.

In this study, SMA is defined as the intake of antibiotics to treat self-diagnosed health conditions without consulting a qualified HCP and without any medical supervision. While the expression “pattern of self-medication with antibiotics”, is defined as the way participants requested and obtained antibiotics for self-medication, to use without a written prescription from a qualified health care professional (HCP).

### Customers study tools

To access the SMA patterns from the customers perspective, data were collected through in-depth face-to-face interviews and FGD. These tools were combined to reach the central characteristics of the phenomenon across different participants in the individual and group perspective to enrich the data and enhance the trustworthiness of findings [20]. Both interviews and FGD guides for customers were based on the objectives of the study and consisted of demographic characteristics (age, gender, education level, profession), knowledge about antibiotics use and resistance, attitudes and behaviors towards antibiotics use, patterns and reasons for SMA, as well as sources of antibiotics. Definition of antibiotics, self-medication, antibiotic resistance as well as the list of antibiotics being officially used within the country were included.

### Pharmacists study tools

Pharmacists’ data regarding the way customers request NPA and the practice of SMA was assessed by using in-depth face to face interviews only. FGD was initially planned but no pharmacist agreed to participate due to the fear of identifications and its implication for their work and the job posts. The interview guide included demographic characteristics of participants (age, gender, education level, years of experience), current practices of SMA, description of activities at the pharmacy, commonly used antibiotics for SMA, routinely dispensed NPA. Additionally, it looked at the knowledge about antimicrobial resistance, pharmacist‘s role and the perceived factors influencing the SMA behavior, the challenges and barriers they face to dispense antibiotics and their suggestions towards the better use of the antibiotics.

### Data management and analysis

The interviews and FGD were audio-recorded, transcribed verbatim and the transcripts were coded and thematically analyzed using latent content analysis. Themes derived from the data, since factual data coding was performed, and categories and sub-categories were identified. The lead researcher read the transcripts to familiarise herself with the data and made analytical notes to inform the coding stage. Data were coded by two coders. During coding, a selection of transcripts was read line by line and initial labels or ‘codes’ applied to each passage that described the essential meaning of the data within. The coding tree included the main question, the answers of participants and the themes and subthemes extracted. Words used, context, internal consistency, the specificity of responses, and overlying themes were considered. Themes emerged from the analysis included reasons for SMA, factors influencing, knowledge of antibiotics, main sources of antibiotics and patterns of SMA, the last one presented and discussed in this manuscript. Nvivo software (version 11) was used to store and retrieve the data.

## Results

### Demographic characteristics of customers and pharmacists

Table 1 shows the demographic characteristics of customers with variation in age (range:19–67), education level (range from primary to a university degree), and gender (with 22 women and 10 men participants). Table 2 shows the demographic characteristics of the pharmacists with variation in age (range: 24–47), professional training and years of professional experience (range: 1–12).

**Table 1:** Demographic characteristics of customers (*n* = 32)

Variable	Character	Frequency (n)	Percentage (%)
Age in Years	19–20	3	9.4
	21–30	10	63
	31–40	9	28
	41–50	6	19
	51–67	4	13
Gender	Female	22	68,8
	Male	10	31,2
Level of Education	Primary/Basic	7	21,8
	Secondary	16	50
	University Degree	9	28,1

**Table 2:** Demographic characteristics pharmacists (*n* = 17)

Variable	Character	Frequency (n)	Percentage (%)
Age in Years	24–34	4	23,5
	35–44	10	58,8
	45–47	3	17,6
Gender	Female	11	64,7
	Male	6	35,2
Professional Training	College degree	8	47
	University Degree	9	52,9
Professional Experience	1–5 years	10	58,8
	6-12 years	7	41,2

Participants who purchased and administered antibiotics without prescription during the last 3 months before the interviews were included. Customers consisted in men 10 (31%) and women 22 (69%) ranging in age from 19 to 67 years old. The average age was 35 years old. The researcher identified four levels of education: primary (1st–7th graders), secondary (8th–12th graders), first degree (university graduate level). Seven participants (22%) had basic education level, half had secondary education level (50%), while eight (25%) had a first degree and one participant had master’s degree (3%). Pharmacists demographics such as gender, age, the level of training and years of working experience were accessed and presented in Table 2 below.

### Patterns of SMA

Self-treated customers admitted having obtained their antibiotics using the following patterns: a) at the pharmacy by referring to their scientific/generic or brand names; b) at the pharmacy referring to physical appearance or the empty package of the antibiotic previously used; c) at the pharmacy after seeking advice from the pharmacist by describing the symptoms and health condition; d) at the pharmacy by referring to an old and not valid prescription; e) by sharing prescriptions or the antibiotics itself with family, friends or neighbors. Each identified pattern is described beneath. The quotes from the participants are presented according to their socio-economic areas, high socio-economic area (HSE), middle socio-economic area (MSE) and low socio-economic area (LSE).

#### Obtaining NPA by referring to their scientific/generic or brand names

Purchasing antibiotics by referring to the scientific or generic name was one of the most common patterns of SMA used by the participants from the middle and high socio-economic pharmacies. The names of antibiotics were known by the participants since most indicated previous use of, prescribed by an HCP when they had sought help at the hospital or clinic. Participants who requested an antibiotic using this pattern named amoxicillin with clavulanic acid, azithromycin, and cotrimoxazole as the most purchased NPA. Although participants did not know precisely the uses and adverse effects of the requested antibiotics, they mentioned the antibiotics were useful in treating the health condition in 2 to 3 days as shown in the quotes:

“I got the prescription before when I had to seek for a medical appointment! hmmm…this time I got the same difficulties swallowing, so I came to buy more last week… but they are almost finishing so I need more of this (antibiotic- azithromycin)…” *(individual interview, customer 1, HSE pharmacy).*

“Well, I bought cotrimoxazole its good for cough…in two days I will be fine” *(individual interview, customer 3, MSE pharmacy).*

“I know these tablets, I went to a doctor before…months ago, he gave me the prescription, these antibiotics (amoxicillin with Clavulanic acid) are good (…)” *(individual interview, customer 2, HSE pharmacy).*

“…at the pharmacy I just asked for this hmm…I know this medicine (amoxicillin with clavulanic acid), I had an abscess in the past and the doctor gave me this. (*individual interview, customer 11, HSE pharmacy).*

“We live in a very dusty environment, so time to time we have a cough and have blocked nose… going to the hospital every time is time-consuming and you face queues just for coming up with a prescription of syrups… I just bought cotrisha (cotrimoxazole tablets), they treat cough very well” *(individual interview, customer 17, MSE pharmacy).*

When questioned the manner customers request NPA, pharmacists unanimously admitted that SMA are frequent practices among customers. One pharmacist admitted SMA is a widespread practice and that most customers seem to be very well informed:

“SMA practices are frequent…yes, that is a normal practice nowadays…people are informed...I don’t know to what extent…laughs. But also, because there are many situations in which the patient presents claims and symptoms to the pharmacist, the pharmacist thinks that he needs to help the patient, pause…apart from this help, there are also many patients that ask directly to antibiotics... *(individual interview, pharmacist 1, HSE pharmacy).*

According to the pharmacists, most customers know exactly the name and dosage of the antibiotics (how many milligrams, e.g. If 250 mg or 500 mg) and occasionally they refer to the name of the laboratory of origin of the antibiotics (if a Germany, Portuguese or Indian made antibiotic):

“most of the times it is scary…really scary hmm. Because the patient or customer gets into the pharmacy and just ask for amoxicillin with clavulanic acid and he makes sure you don’t give them the Indian one, as they say, it has more side effects that the Germany or Portuguese one…it is really difficult (…) hmm in these cases me I’m just a medicine vendor” *(individual interview, pharmacist 5, HSE pharmacy).*

“You know, nowadays with access to internet things are getting complicated for us… you know… a customer coming to your pharmacy and requesting amoxicillin 250mg without a prescription. They know exactly what they want and for what…and when they have the money, they even tell you the brand (laboratory) they want” *(individual interview, pharmacist 3, MSE pharmacy).*

Although pharmacists agree SMA is an individual practice, they believe the doctors and/or qualified health professionals are also to blame for the massification of the use of antibiotics since they are quick to prescribe for symptoms or health problems that do not necessarily require antibiotic treatment. Moreover, pharmacists believe doctors’ prescribing practices contribute to SMA since they frequently prescribe the same antibiotic for different conditions and patients learn to recognize those antibiotics. This according to pharmacists lead patients to overuse and don’t feel the need to seek medical help since patients who can afford to pay, go straight to the pharmacy:

“Doctors should know that patients are not blind, they can think, they talk to each other, they share information. Some doctors prescribe the same antibiotic for 2 or three patients, those patients met at the hospital pharmacy trying to buy the same medicine but they have some different symptoms and we know…most of the time at hospital facilities they have a shortage of some antibiotics so because our pharmacy is here close to the hospital those patients end up here…you can see they have the same prescribed antibiotics…next time they got sick they just don’t go to the hospital they came straight here…see” *(individual interview, pharmacist 13, MSE pharmacy).*

“The knowledge people have about some antibiotics come from previous experience…something is wrong with prescribers…they are the ones marketing this, apart from the internet. Doctors they don’t even listen to the patients I think…they just prescribe amoxicillin or azithromycin (…)it is their behavior I know… but it comes from somewhere” *(individual interview, pharmacist 10, MSE pharmacy).*

“Patient seeks medical help at the hospital or clinic, once, then twice, doctor or nurse prescribes the same antibiotic for any pain amoxicillin, for any cough…cotrimoxazole, so people get confidence in a certain antibiotic, they take ounce and see good results…them take twice and feels ok, that patient will not stop. I don’t know if it is just fancy to prescribed antibiotics.” (*individual interview*, *pharmacist 8, HSE pharmacy).*

#### Obtaining NPA by referring to the physical appearance or to the empty package of antibiotics

Mentioning the color, the shape and the size of the drug to describe antibiotics such as amoxicillin, tetracycline, and cotrimoxazole was found to be common among participants from the MSE area of the city. Most participants reported they have known amoxicillin as the *“two colors medicine”* describing the capsules which normally come in red and yellow or red and white colors. They also mentioned *“the yellow capsules to treat wounds”,* referring to tetracycline capsules that customers open and use the powder content:

“I have this sinus problem, so there are some tablets, I don’t remember the name, but they have a name, well they are used to treat my rhinitis problem, they are yellow and red they are very good to my problem, for me when I take this drug they just cut the rhinitis” *(individual interview, customer 4, LSE pharmacy).*

“My firstborn had some wounds in the head…he got from other kids at the neighborhood, I went to the hospital with him, stayed there all day and nurse gave a skin cream and *savlon* (antiseptic liquid) to wash the wounds but did not work, so my neighbor gave me one tablet to try and use the powder to cover the wound…it has gone…now I’m buying more of this for the other boy” *(individual interview, customer 9, MSE, pharmacy).*

The interviewees who reported this pattern could not name the antibiotic but could describe the appearance of the drug correctly and recognize it by the size and color. Moreover, participants admitted those antibiotics were prescribed for them or someone else for a past event of sickness.

“I was prescribed initially, but I lost the script. I always purchase this here at the pharmacy, I ask the pharmacist for “two colors” tablets used to treat inside wounds” *(individual interview, customer 14, LSE pharmacy).*

“One day there was a new pharmacist and I requested the “two colors”, he gave me white and red, while I normally use the red and yellow…I took those for 2 days and was not feeling better…the yellow ones are very good even for fever…” *(individual interview, customer 4, LSE pharmacy).*

“I went to remove a tooth month ago…and the dentist gave me the prescription for this I know them, but my sister said they are also good for this pain I have here I feel like burning when I go to wee, so I came to buy 10 of this” *(individual interview, customer 15, LSE pharmacy).*

Participants from one FGD not only admitted requesting non-prescribed drugs by showing the empty package of previously used antibiotics but also admitted that showing the empty package helped to make sure the pharmacist dispenses the right antibiotic and to convince pharmacists that they were under treatment:

“When you finish treatment with the tablets it is not good to throw away the package as it may help to buy more in case you need to continue with treatments you won’t get the wrong tablets and pharmacist can see you are under treatment” *(Customers FGD 1)*.

#### Obtaining NPA after describing the symptoms and health conditions to the pharmacists

The pharmacists stated they usually advise customers on what antibiotic to take when asked for assistance. Although such advising practice is not part of the pharmacist’s role, pharmacists believe that they are bound to help customers seeking assistance.

“Some customers are very smart…they don’t seek medical aid at the hospital, they came straight to your pharmacy and ask for advice…like they feel bad…some of them are really bad you can see, so you can’t deny assistance for them, we should help them with medicines to relief and tell them to go to the hospital afterward…” *(individual interview, pharmacist 12, MSE pharmacy).*

Customers considered the advice provided by the pharmacists had a significant impact on their health outcomes. These customers perceived pharmacists as health workers and therefore entitled to advise and/or recommend what medicine should be administered for a determined health condition. Thus, pharmacy customers generally experienced very little difficulty in purchasing certain antibiotics especially the well-known antibiotics such as amoxicillin, cotrimoxazole, tetracycline, and azithromycin:

*“*I just arrive at the pharmacy and explain to the pharmacist what I feel, the symptoms, and they just advised me on what antibiotic to purchase, the direction to take them… they are very helpful “(*individual interview, customer 3, HSE pharmacy).*

“When I feel sick… I just come to the pharmacy, the lady here is very good she can help us…I just tell what I feel yeah…this time my ear is painful, and she advised me to use these drops” *(individual interview, customer 19, MSE pharmacy).*

“Me…laughs… I can’t imagine my life without these pharmacies all over the city…I’m a busy person and time is limited, sometimes I feel sick at night, I don’t bother, I come and explain what my problems are…” *(individual interview, customer 18, HSE pharmacy).*

*“*although some of them when you open yourself, they advise you to go to the health care center…but, not all are like that…pause, you just explain your health problems to the pharmacist, they are also health workers are not them?! They studied for that…it helps*” (Customers, FGD 2).*

“To purchase medicines without the paper from the hospital??…laughs…it is not that difficult never had problems I don’t know the others at least the “two colors“(…)that’s not difficult.” *(Customers, FGD 1).*

The availability and accessibility of private pharmacies, as well as the pharmacists’ advice, was perceived by the customers as a relief and a time saver since they consider the access to public HCF difficult and time-consuming. The quote below elucidates:

“Most of the times you just get to the public hospital to find out that the nurse or doctor is late, or you wait forever to be assisted when assisted you get the prescription of for example paracetamol, Clavamox and some other drugs you go to the hospital pharmacy and they are out of stock…you should go buy outside private pharmacies are simply a good thing” *(individual interview, customer 17, HSE pharmacy*).

#### Obtaining NPA by using old prescriptions

Old prescriptions are referred to those prescriptions that were written in the past by a doctor or a qualified health professional for certain symptoms health condition after a set of procedures and observation. In this study, prescriptions were considered old if had more than 15 days after prescription. Copy of the prescription is kept back by the patients in Mozambique for reference only. Nevertheless, Patients bring back or refer to the previous prescription to ask for the same antibiotic for similar symptoms for themselves or a different patient, mainly family members or friends. Although it is not permissible legally, pharmacists generally dispense antibiotics on old prescriptions. In this study, three participants had old prescriptions that they used to obtain the antibiotic. Two of them argued:

“The nurse gave me this script more and less 2 months ago and she recommended me to take for 7 days…I had terrible flu with fever, it worked very well.” *(individual interview, customer 5, MSE pharmacy).*

“I went to the doctor not long ago, don’t remember the dates but less than 2 weeks, this prescription is from that time, I could not buy all the tablets, money was not enough …laughs (…)” *(individual interview, customer 12, LSE pharmacy)*.

Similarly, pharmacists admitted with concern, that some customers use old prescriptions to purchase antibiotics. According to the pharmacists, it is also becoming common for customers to come with an old prescription as a picture saved in their smartphone or even shared by someone else. Three pharmacists explained:

“Because they kept back the copy of the scripts, you find customers requesting medicines with those copies of old prescriptions, and you can see that this was already dispensed …if you go deep you find out that the prescription is not under the name of the customer that appears to you…but they can convince you, they can argue that… When a person wants that antibiotic, he tries all…” *(individual interview, pharmacist 7, MSE pharmacy).*

“Normally old patients, people above 60 years old they use a lot of old prescriptions, they can keep it very well and come later to purchase the same medicine…with many arguments and excuses, we are now used to it… hmm but young people they save copies in the smartphones or they have it as a picture, then they came and show me the script in their phone and asks (…)” *(individual interview, pharmacist 1, HSE pharmacy).*

“(…) internet is doing its part in this process…looks like we are all health care professionals now, and it looks fine until you get into troubles…as I said before, this is scary! Educated people come here and show me a script on their smartphones. So, one goes to the hospital and the rest just share the scripts since they think the symptoms are the same…” *(individual interview, pharmacist 3, MSE pharmacy).*

#### Obtaining NPA from family members, friends or neighbors

Both customers and pharmacists mentioned the practice of sharing antibiotics with family, friends and/other people from the social network is common. Such behavior is, according to the pharmacists, influenced by the behavior of not finishing the complete course of treatment and/or stopping medications when symptoms disappear. This attitude leads patients to do home storage of leftover antibiotics that are later used either for the same patient or others within the social network or family. Participants from FGD, customers and a pharmacist agreed and explained:

“Sometimes they say 7 days of antibiotic intake…but one can forget especially when you start feeling better, you stop the medication, it happens to all of us…laughs.” *(Customers, FGD 1).*

“The remaining antibiotics, leftovers? I don’t throw it away…medicines are precious stuff I keep them in my drawer (…)”. *(individual interview, customer 18, MSE pharmacy)*.

“I had these capsules, the “two colors“, a friend gave me for my pain, I was having difficulties to urinate, it was painful…I took it and it was gone” *(individual interview, customer 16, LSE pharmacy).*

“People share a lot of antibiotic and antidepressives prescriptions, for antibiotics, they share prescriptions and even the antibiotics itself, hmmm Clavamox, azithromycin… the ones for sexually transmitted diseases it is common…doxicilinn, metronidazole vaginal cream…HIV positive patients…oh yeah, they tend to share antibiotics and prescriptions” *(individual interview, pharmacist 6, MSE pharmacy).*

“…so, my neighbor gave me one tablet to try and use the powder to cover the wound…it has gone…” *(individual interview, customer15, LSE pharmacy).*

## Discussion

Previous situation analyses from GARP-Mozambique identified strategies to improve the use of antibiotics and contain AMR within the country. The report emphasized the need to reduce the inappropriate consumption of antibiotics in the community and recommended urgent studies that could evaluate the patterns of antibiotic use at the community level [17]. However, to date, no published research has investigated the patterns of SMA among customers although such knowledge is critical to formulating appropriate interventions to improve the current situation. This is the first qualitative descriptive study exploring patterns of SMA by describing the models and ways in which pharmacy customers access, request or purchase NPA, their perceptions and attitudes in Maputo city, Mozambique.

The Mozambican medicine law number 12/2017, endorses that all medicines and health products should be dispensed with prescription except the ones classified as non-prescription medicines which is not the case of antibiotics. In Mozambique, all medicines purchased in the pharmacies of the public and private HCF must be prescribed by medical doctors, physicians or qualified HCP. In the public health facilities and its pharmacies, all medicines are provided against prescription only, at a very low cost to patients because they are subsidized by the government. At private pharmacies, patients/customers pay the stipulated amount for medicines. Majority of the population cannot afford to pay for medicines in the private pharmacies, but public-sector pharmacies frequently lack stock of some medicines [21]. In such cases, the patients pay more for their medication at a private pharmacy.

Langdon (1995) supports the idea in which self-medication is conceived as a process of self-care from the biomedical and cultural point of view [22]. SMA is perceived as an expression of self-care when participants experience self-perceived symptoms and indulge in self-treatment as a method of caring for themselves and the people within their network. For Geest and Whyte (1989), medicines are an important part of the process of self-care and self-treatment as they are the core of therapeutic actions [23].

The need for individual self-care associated with the healing power ascribed to antibiotics [24], leads participants to indulge in self-diagnosis and self-treatment practices. The practice of SMA suggests a widespread set of beliefs related to the perceived need to use antibiotics regardless of prescription. This is concordant to the research conducted by Gartin et al.,(2010), which reported that in Mexico pharmacies customers believed the use of antibiotics regardless of prescription is their right since they are intitled of their health [24].

The views put forward here suggests that the patterns in which customers from different socio-economic groups access NPA to treat self-diagnosed health conditions are certainly contributing to antibiotic misuse, abuse, and to the increase of antibiotic resistance. The views also reveal that socio-economic and cultural factors are affecting health-seeking behavior and the non-compliance with medication courses. The patterns of SMA reported in this study are concordant with the classification of Foster and Bandawe (2014). The results show participants indulged in both: a) direct self-medication - referring to scientific/generic names, brand name or physical appearance, use of leftover antibiotics from previous disease, presentation of empty previous drug package; b) indirect self-medication - seeking advice from the pharmacist, referring to an old and not valid prescription and sharing prescriptions or the antibiotics itself with family, friends or neighbors.

The explanation people make for a given experience of illness and the therapeutic itineraries they adopt are resultant of the different means by which they acquire their medical knowledge [22, 25]. According to the results, participants perceive antibiotics as marvelous medicines that can treat “everything”. This consequently led to request and/or purchase antibiotics based on previously experienced treatment. Since participants looked to alleviate symptoms and not necessarily to fight the bacteria, in most of the cases the health conditions treated with the accessed NPA were not bacterial infection. This can be explained by Alves (1993) who noticed that the medical knowledge of an individual always has a particular history since it is constituted by diverse experiences [25].

The indirect pattern of SMA that consisted of participants sharing antibiotics, shows that participants may have received the prescription and advice of a doctor or HCP authorized to prescribe. Though, when the symptoms re-appear or a member of the family or a friend has the same symptoms, people tended to indulge in self-medication practices by acquiring or sharing the same medication. Similar practices were reported by Kotwani et al., (2012) and Viberg et al., (2007). This paves the way to the occurrence of recurrent infections due to deficient treatment. Therefore, education strategies are needed to raise awareness, discourage the sharing of medicines and deconstruct the belief that antibiotics are effectives to treat all types of diseases. Similarly, refreshment training for physicians and HCP, re-educating on the differences between viruses and bacteria in the etiology of upper respiratory infections would probably be effective to prevent the excessive prescription of antibiotics for viral infection.

Pharmacists agreed they dispense NPA to customers frequently, especially when there is weather transition and or changing from winter to summer or vice versa as in this period some health problems such common flu, cough, sore throat, and rhinitis are more likely to raise, and people seek help at the pharmacy. The pharmacist recognized that antibiotics are amongst the most pharmaceuticals’ products sold after analgesics and anti-inflammatory drugs. For them, the high demand for antibiotics is also related to unappropriated prescriptions since most of the time physicians and HCP prescribe antibiotics needlessly for non-bacterial health conditions. This according to the pharmacists, led customers to get used to those antibiotics and start purchasing even without a prescription. These results are in line with the ones reported by Kotwani et al., (2012), in an Indian context, where pharmacist complained that community is demanding for more NPA in the same way doctors are prescribing them [26].

This study adds to the growing body of knowledge the need to develop effective education and health promotion strategies to deal with all sort of inappropriate use of antibiotics including the SMA practices. Thus, all sort of interventions that include physicians, patients, pharmacy customers and pharmacists should be considered to improve the rational use of antibiotics in-country.

### Limitations of the study

Our findings contribute to the general understanding of uncontrolled antibiotic use in Maputo. Although there is a need for extensive research on the patterns of SMA, as well as on the prevalence rates, this study may serve as exploratory evidence basis for understanding community practices and pharmacist’s compliance regarding the appropriate utilization of antibiotics in this setting. Despite presenting a comprehensive summary of the phenomena of SMA, limitations of this study include those known for qualitative descriptive studies. For example, despite the number of pharmacies included in the study guaranty differences in terms of the socio-economic characteristics of the customers, these pharmacies are not representative of all pharmacies in Maputo. Another limitation is the fact that the practices, the reasons and the behaviours regarding SMA were more important to describe rather than the frequencies and percentage of each pattern which would give a larger extension of the problem.

### Implication for practice and future research

In terms of the policy, health authorities must address the problem by considering different strategies and educational programs for customers to raise awareness on the disadvantages and side effects of utilization of NPA including bacterial resistance and the high financial cost for the health system. At the same level, refreshment training for HCP including pharmacist are urgent to address the inappropriate prescribing, dispensing and the non-compliance to law and regulations. This should be in parallel with reinforcement of the existing law and protocols for antibiotic prescribing and dispensing. Future research would be more beneficial if consider larger qualitative and quantitative studies on the patterns of SMA involving households to include frequencies and percentage of each pattern.

## Conclusion

The study comes to the conclusion that pharmacy customers from Maputo city suffer inappropriate usage of antibiotics since different patterns of SMA are well-rooted. Customers take advantage of biomedical knowledge and use it in their daily lives, leading SMA to be a common practice which occurs regardless of the socio-economic area, age, and education level and it is perceived as an expression of self-care. Customers experience self-perceived symptoms, they do self-diagnose and indulge in self-treatment as a method of caring for themselves and the people within their social network. This is done with the consent of the most knowledgeable practitioner on the health risk of SMA; the pharmacists who dispense the NPA.

The misunderstanding of antibiotics as antitussives, painkillers or antipyretics has been affecting the suitable use of these drugs among customers. This thus symbolizes health risks for them while the pursuit of profits is driving the pharmacist practices and undermining the health risks for pharmacies customers. Strong and effective public health education and promotion initiatives should be implemented to discourage inappropriate utilization of antibiotics and SMA practices. Also, professional regulatory and law enforcement of the already approved dispensing guidelines and quality assurance processes should be considered.

## Data Availability

The raw data was attained in Portuguese language, the datasets were translated and transcribed to English. The data are not publicly available as it contains information that could compromise research participant privacy/consent. Therefore, the datasets analyzed for this paper will be assessed from the corresponding author upon rational request.

## Abbreviations

AMR: Antimicrobial resistance
HCF: Health Care Facilities
HCP: Health Care Professionals
ISCISA: High Institute for Health Sciences
LMICs: Low and Middle-Income Countries
MoH: Ministry of Health
MRSA: Methicillin-Resistant *Staphylococcus aureus*
NPA: Non-prescribed antibiotics
OTC: Over- the -Counter
POM: Prescription -Only Medicine
SMA: Self-Medication of Antibiotic
UKZN: University of KwaZulu-Natal
WHO: World Health Organization

## Footnotes

1 prescription with not more than 7 days after being prescribed by a physician

2 The medium level, normally takes 2 to 3 years training in a technical school.

3 The university level, which take 4 to 5 years training in a university.
